# CNN-Based Spectral Super-Resolution of Panchromatic Night-Time Light Imagery: City-Size-Associated Neighborhood Effects

**DOI:** 10.3390/s21227662

**Published:** 2021-11-18

**Authors:** Nataliya Rybnikova, Evgeny M. Mirkes, Alexander N. Gorban

**Affiliations:** 1 Department of Mathematics, University of Leicester, Leicester LE1 7RH, UK; em322@leicester.ac.uk (E.M.M.); a.n.gorban@leicester.ac.uk (A.N.G.); 2 Department of Natural Resources and Environmental Management, University of Haifa, Haifa 3498838, Israel; 3 Department of Geography and Environmental Studies, University of Haifa, Haifa 3498838, Israel; 4 Institute of Information Technologies, Mathematics, and Mechanics, Lobachevsky University, 603105 Nizhny Novgorod, Russia

**Keywords:** night-time light (NTL), panchromatic, red, green, blue (RGB) bands, international space station (ISS), convolutional neural network (CNN), neighborhood effect

## Abstract

Data on artificial night-time light (NTL), emitted from the areas, and captured by satellites, are available at a global scale in *panchromatic* format. In the meantime, data on *spectral* properties of NTL give more information for further analysis. Such data, however, are available locally or on a commercial basis only. In our recent work, we examined several machine learning techniques, such as linear regression, kernel regression, random forest, and elastic map models, to convert the panchromatic NTL images into colored ones. We compared red, green, and blue light levels for eight geographical areas all over the world with panchromatic light intensities and characteristics of built-up extent from spatially corresponding pixels and their nearest neighbors. In the meantime, information from more distant neighboring pixels might improve the predictive power of models. In the present study, we explore this neighborhood effect using convolutional neural networks (CNN). The main outcome of our analysis is that the neighborhood effect goes in line with the geographical extent of metropolitan areas under analysis: For smaller areas, optimal input image size is smaller than for bigger ones. At that, for relatively large cities, the optimal input image size tends to differ for different colors, being on average higher for red and lower for blue lights. Compared to other machine learning techniques, CNN models emerged comparable in terms of Pearson’s correlation but showed performed better in terms of WMSE, especially for testing datasets.

## 1. Introduction

Artificial night-time lights (NTL), emitted from the residential, industrial, and entertainment areas, and captured by satellites, provides researchers and policy-makers with the information for a wide range of analyses: on the human presence on the Earth [1,2,3,4,5,6,7,8], on NTL adverse effects on human health [9,10,11], on the health of ecosystems [12,13], on night sky observations [14,15,16], etc. Globally, this NTL information is currently provided by the day-night band (DNB) sensor, supported by Visible Infrared Imaging Radiometer Suite (VIIRS), and available from the Earth Observation Group site [17]. These data, however, are panchromatic (that is, each pixel of NTL image reports summarized intensities of light in the diapason of 500–900 nm [18]). In the meantime, information about NTL *color* is of great importance for a variety of research, since it is known, for instance, that NTL emissions of different diapasons are associated with different economic activities and land-use types [19,20,21], or that NTL in blue diapason is especially effective in melatonin suppression [22] and thus inducing hormone-dependent cancers [23] and obesity [24]. Red, green, and blue (RGB) spectrum of NTL data, however, are currently available from the International Space Station (ISS) for selected areas and sporadically [25], or on a commercial basis [26], thus impeding research in a variety of fields. The present study proposes an approach to obtain RGB NTL information, highly demanded the epidemiological and nature conservation research from freely available panchromatic NTL and auxiliary built-up area data.

In our previous research [27], we attempted to obtain RGB NTL estimates from freely available panchromatic VIIRS-DNB NTL data and information on built-up areas, used as land-use type proxies. We used several machine learning techniques, such as linear regressions, non-linear kernel regression, random forest, and elastic map models to define the association between ISS-provided RGB NTL levels at certain pixels and a set of predictors, comprising panchromatic NTL and built-up areas characteristics from corresponding pixel and its *first-order neighbors*. We built such models for each of the eight metropolitan areas all over the world—Atlanta (US), Beijing (China), Haifa (Israel), Khabarovsk (Russia), London (UK), Naples (Italy), Nashville (US), and Tianjin (China) —and validated them over the rest seven metropolitan areas. The analysis showed that model-estimated RGB NTL levels demonstrated a sufficient consistency with the original ISS-provided RGB NTL data: Pearson’s correlation coefficients, both for training and testing sets, ranging between 0.719 and 0.963, and weighted mean squared error (WMSE) varying from 0.029 to 4.223. This study considered the first-order neighbors of each point only. In the meantime, accounting for the information from *more distant neighboring pixels* (than first-order ones) might be beneficial for obtaining more accurate estimates of RGB NTL levels of the pixel under analysis. The reason is that certain land-use types within a city (residential areas, industrial facilities, municipal roads) may differ in physical size, NTL intensity, and spectral characteristics [19,20,21]. In the present study, we aim at assessing this neighborhood effect. 

Since we aim at enhancing the spectral resolution of NTL images from panchromatic to RGB, our task might be viewed as a super-resolution (SR) task, which refers to the process of recovering high-resolution images from low-resolution ones [28]. SR, which initially referred to the *spatial aspect* of the images only, is nowadays one of the most active research areas [29]. Spatial SR research utilizes either frequency-domain-based, interpolation-based, regularization-based signal processing techniques, or machine-learning methods (for reviews, see [30,31,32]). Recently, with the development of deep learning techniques, convolutional neural networks (CNN) have widely been utilized in spatial SR research (see, for example, [33,34,35,36,37,38]; for review, see [28]). In line with spatial, *spectral SR research*, modeling the mapping from coarser scale spectrum (usually, RGB) to finer one (usually, hyperspectral images of over 30 spectral channels) [39], is also nowadays quickly developing. Compared to spatial, spectral SR research is much more sparse [40] mainly due to the lack and incomparability of training data: hyperspectral cameras are expensive and generally do not simultaneously produce broader RGB bands–thus, low and high spectral resolution images vary in terms of the scene. Additionally, spectral responses of sensors are camera-specific and may vary significantly from camera to camera [41]. Thus, recent studies on spectral SR utilize hyperspectral images, comprised of ~30 bands from 400 to 700 nanometers with 10 nm increment, from the ICVL (201 images), CAVE (32 images), and NUS (66 images) datasets (see, for example, [40,41,42,43]). Probably due to later start, spectral SR studies are often based on neural networks, either shallow (see, inter alia, [42,43,44]), or moderately and very deep (see, inter alia, [40,41], correspondingly) CNNs. A shallow neural network approach implies using a high-quality hyperspectral dataset to create a sparse dictionary of hyperspectral signatures and their RGB projections and afterward to restore a hyperspectral image from decomposed–over the dictionary–pixels of a new RGB image [42,43,44]. This approach is reported (see [43]) to be much faster in computation, but comparable in terms of performance with its predecessor, based on the matrix factorization method [45]. Simultaneously, a shallow neural network approach was reported (see [42]) to be similar in performance in terms of RMSE to very deep CNN, with a total of 56 layers [41]. The reason might be in the lack of training data and high correlation between spectral bands, which require a moderately deep CNN; as a recent study confirms (see [40]), such a CNN, consisting of 6 layers only, outperforms both shallow and very deep CNN. 

Despite the formal proximity to the *image colorization task*, also–as in our case–implying enhancing the spectral resolution of the image from grayscale to RGB [46], our task is essentially different. In image colorization, mainly applied to historical photographs and videos, the research, aimed at diminishing the user input, is generally based upon finding an appropriate reference image (or selected semantic feature) from a dataset and transferring its color to the whole or the part of the image under analysis [47,48,49,50,51,52]; the research is built upon semantic features (such as cars, animals, plants, etc.) identification and allocation.

In essence–not formally–our task is different from the image colorization task. Unlike the latter, our research does not imply user intervention or semantic features identification. Actually, our task is a spectral SR task. The difference is that we deal with night-time satellite images, which, due to low signal, are not available in the hyperspectral format, and it is a great privilege to obtain even RGB resolution. Given the ill-posedness of the task of mapping between coarser and finer spectral resolution [39,40,41,43], in our research, we use built-up area characteristics as additional input information. We hypothesize that panchromatic light and built-up area information from neighboring pixels might contribute to better predictions of RGB levels. Using CNN as a tool, we settle our task as a regression one: we form multi-layer small-size images from panchromatic VIIRS-DNB NTL and built-up area data, which are further used as CNN input images, and match them with the levels of either red, green, or blue light pixels, located in the center of the corresponding small-size image, and used as a dependent variable. We run our analysis upon eight metropolitan areas all over the world. We experiment with the size of input CNN images—we try square images with an odd number of pixels (from 3 × 3 to 15 × 15). We compare the performance of the models overtraining and testing datasets for each CNN input image size and also compare the fit to the actual RGB data of the CNNs with previously used machine learning techniques [27]. As our results indicate, compared to our previous study, under-examined first-order neighborhood effect via linear regression, non-linear kernel regression, random forest, and elastic map models [26], CNN models emerged comparable in terms of Pearson’s correlation, but showed generally better performance in terms of WMSE, especially for testing datasets. We assume that such an improvement was induced by accounting for the varying neighborhood effect. The second important finding of the analysis is that for relatively small metropolitan areas, either in terms of area or population, the best-performing models for any color light band prediction were built for 5 × 5 input image size, while for larger areas the optimal input image size was at least 7 × 7 pixels and varied depending on the color prediction.

## 2. Materials and Methods

### 2.1. Data Sources

**Figure 1:**
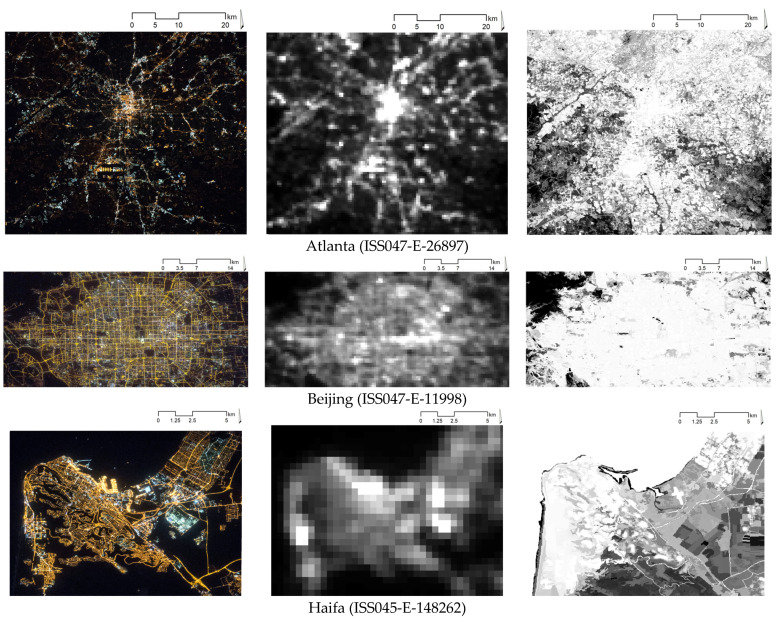

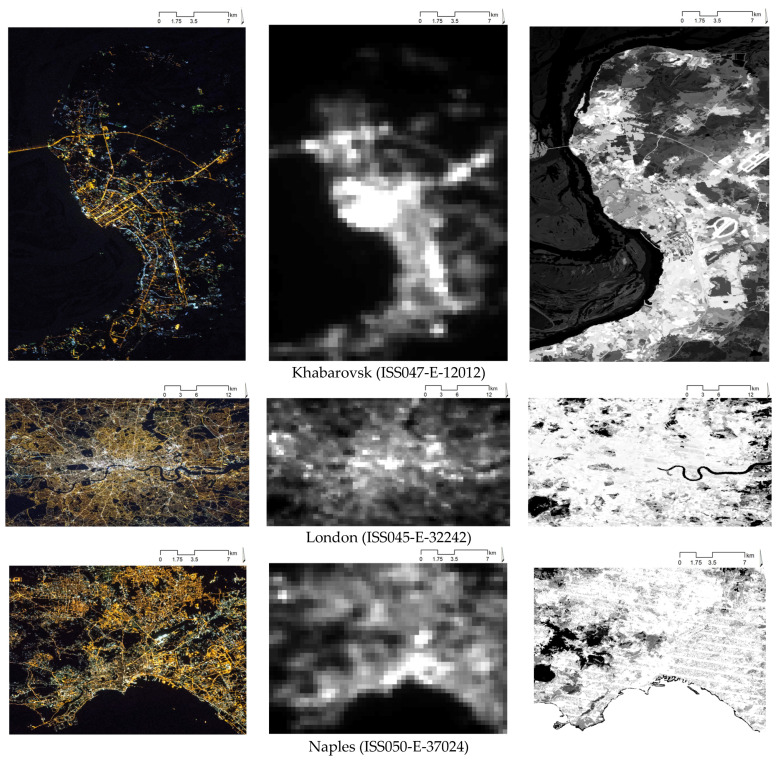

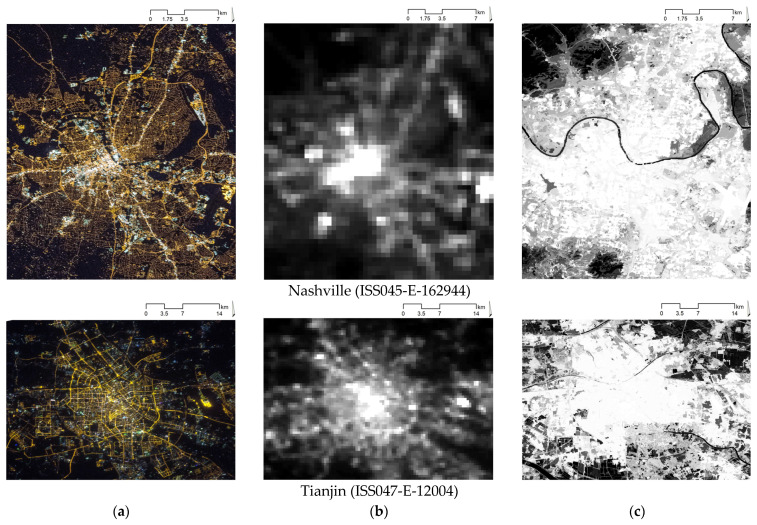
Raw input data for metropolitan areas under analysis: RGB NTL images (**a**), panchromatic NTL images (**b**), and HBASE images (**c**). Notes: RGB NTL images are ~10-m resolution with the range of values of 0–255 digital numbers (dn) for each band; panchromatic NTL images are ~500-m resolution with the values in the range of 1–740 nW/cm^2^/sr; HBASE images are ~30-m resolution with the values in the 0–100% range. For the sake of comparability, RGB images, selected for analysis, were made by the same DSLR camera (Nikon D4), and their acquisition time is close to the VIIRS/DNB acquisition, that is, at about 01:30 a.m., local time.

In the analysis, we use three sources of information. First, as an output of CNNs, we use multispectral (RGB) images, provided by the ISS and available from the Astronaut Photography Search Photo service [25] for eight metropolitan areas all over the world—Atlanta (US), Beijing (China), Haifa (Israel), Khabarovsk (Russia), London (UK), Naples (Italy), Nashville (US), and Tianjin (China). The images and their ID numbers are reported in the left column of Figure 1. 

Second, as CNN inputs, we use panchromatic NTL intensities–namely, we use spatially corresponding cropped areas from VIIRS-DNB-provided images, available from the Earth Observation Group site [17] (see the central column of Figure 1). In the present analysis, to avoid poor-quality pixels, outliers, and cloud contamination, which might be present in daily VIIRS-DNB images [53], we use their monthly composites. 

Third, as additional inputs for CNNs, we use the characteristics of the built-up extent; That is, we use cropped corresponding areas from the global raster layer of human built-up area and settlement extent (HBASE) database, reporting the pixel-wise percentage of the built-up area in the range from 0 to 100%. These data are available from the NASA Socioeconomic Data and Application Center site [54] (see right panel of Figure 1).

### 2.2. Data Processing

**Figure 2:**
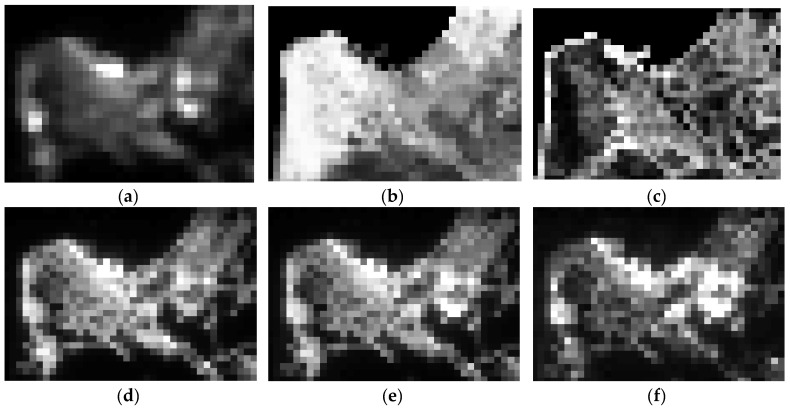
Resampled input data (to the resolution of ~500-m) for the Haifa region (Israel). (**a**) Panchromatic NTL; (**b**) Average levels of HBASE; (**c**) Std. Dev. levels of HBASE; (**d**) Average red light levels; (**e**) Average green light levels; (**f**) Average blue light levels.

To obtain inputs and outputs for neural networks, we used ArcGIS v.10.x software [55] to resample three types of the images (see Figure 1) to the resolution of the coarsest of them—that is, ~500-m resolution of panchromatic NTL data (Figure 2 reports processed images for the Haifa area, Israel). Thus, from the initial HBASE image (right panel in Figure 1), we form two 500-m resolution images, reporting average levels and standard deviations of initial middle-resolution HBASE data in each new coarse pixel (see Figure 2b,c). From each band of the initial high-resolution RGB image (left panel of Figure 1) we form a 500-m resolution image, reporting average levels or red, green, and blue band lights in each corresponding new coarse pixel (see correspondingly Figure 2d–f).

**Figure 3:**
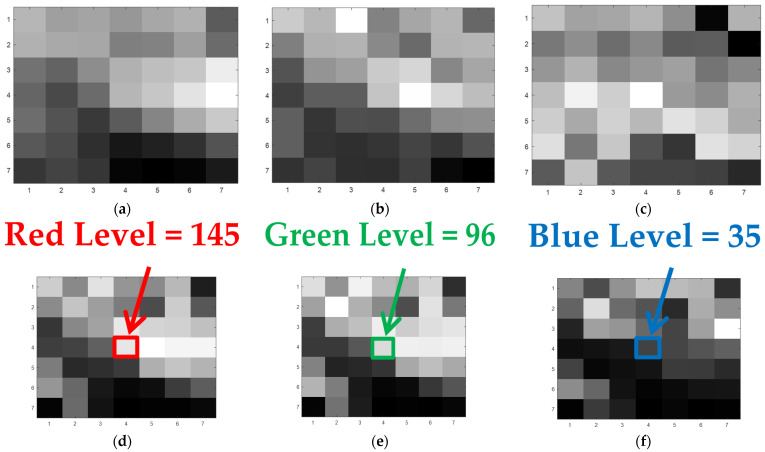
A unit of the CNN input data: Three layers simultaneously used as input images (**a**–**c**) and three color bands with the levels of central pixels (framed) used as alternative output labels (**d**–**f**). Note: The data stand for Haifa (Israel), the central pixel position is the 21st raw, 21st column. (**a**) Panchromatic NTL; (**b**) Average levels of HBASE; (**c**) Std. Dev. levels of HBASE; (**d**) Average red light levels; (**e**) Average green light levels; (**f**) Average blue light levels.

Afterward, using MATLAB v.R2020x software [56], in order to obtain inputs for the convolutional neural networks, we have sliced all the resampled layers (Figure 2) using a kernel with the unit stride into small fragments of *K* × *K* size, Kϵ[3;5;7;9;11;13;15]. Figure 3 reports an example of 7 × 7 fragments of the resampled layers for the Haifa region. At that, fragments of panchromatic NTL, average and standard deviation levels of HBASE (Figure 3a–c) formed three layers of input images for CNN, while the values of central pixels from the red, green, or blue light fragments (Figure 3d–f) consequently emerged as dependent variables.

### 2.3. CNN Architecture

**Figure 4:**
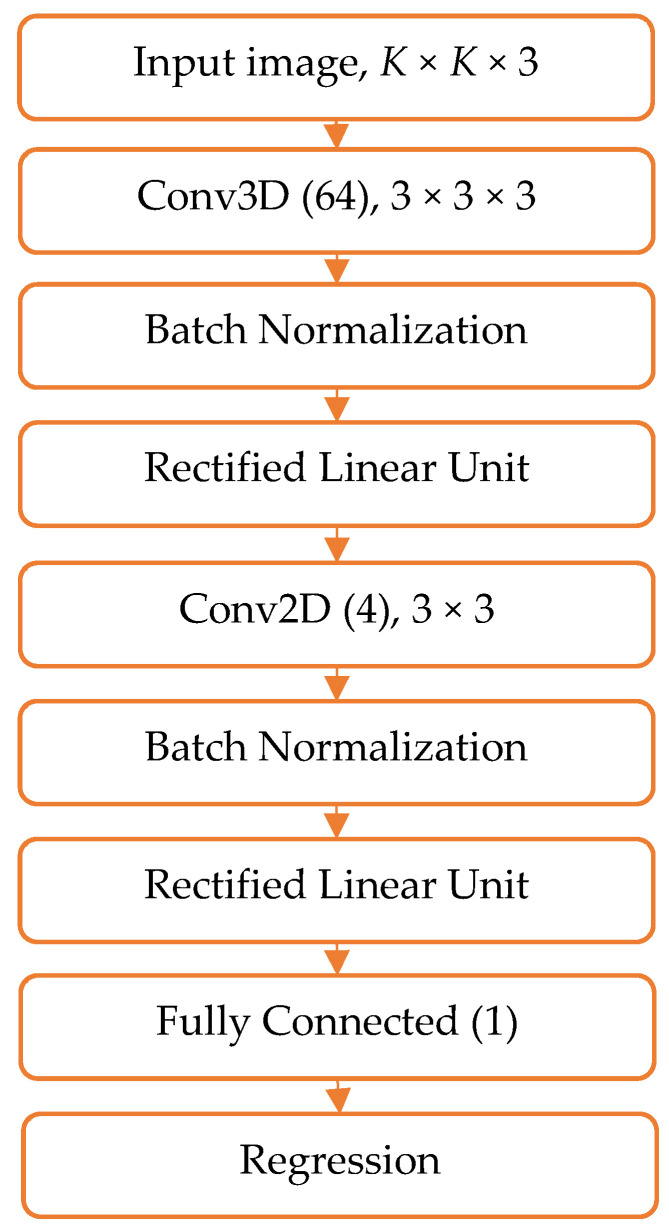
The architecture of the CNN, used in the analysis. Note: Conv2D and followed by BN and ReLu layers were omitted in the case of 3 × 3 × 3 input image size.

Since we had small-scale input images (from only 3 × 3 to 15 × 15 pixels) of a relatively small number (see Table 1), training a heavy CNN seemed not appropriate [57]. Therefore, we performed trial experiments with several ‘light-weight’ CNN architectures and eventually selected the one depicted in Figure 4. The selected CNN architecture included the following consequent layers: (1) Input image layer, represented by the sets of input images of *K* × *K* size, Kϵ[3;5;7;9;11;13;15]; (2) 3D convolutional layer (Conv3D) of 3 × 3 × 3 size, followed by Batch Normalization (BN) and Rectified Linear Unit (ReLu) layers; (3) 2D convolutional layer (Conv2D) of 3 × 3 size, also followed by BN and ReLu layers; (4) one fully connected layer; and (5) regression layer, aimed to associate the output with continuous levels of either red, green, or blue light intensity. 

For either red, green, or blue band light prediction, we trained CNN on the images of a certain metropolitan area and applied the obtained model to each of the rest cities. The analysis was performed in MATLAB v.R2020b software [58] using *TrainNetwork* function [59] with a default training options (i.e., under stochastic gradient descent with momentum (SGDM) optimizer; minibatch size = 128; initial learn rate = 0.001; learn rate schedule = piecewise; learn rate drop factor = 0.1; learn rate drop period = 20; shuffle = every epoch).

### 2.4. Assessing the Quality of the Models

The performance of the models for training and testing sets of images was assessed via two indicators: (i) Pearson’s correlation and (ii) weighted mean squared error (WMSE) between actual and model-estimated red, green, or blue band light intensity. The first indicator is aimed to assess the model’s ability to produce RGB estimates, which in their *relative* tendency correspond well with the observed RGB levels, while the second one, calculated as mean squared difference between the model-estimated and actually observed RGB levels, divided by the actually observed value, helps to assess differences between the estimated and actual RGB levels on an *absolute* scale. Statistical analysis was performed using JASP v.0.14.x software [60].

## 3. Results

### 3.1. Neighborhood Effect: Models’ Performance upon Training and Testing Datasets

**Figure 5:**
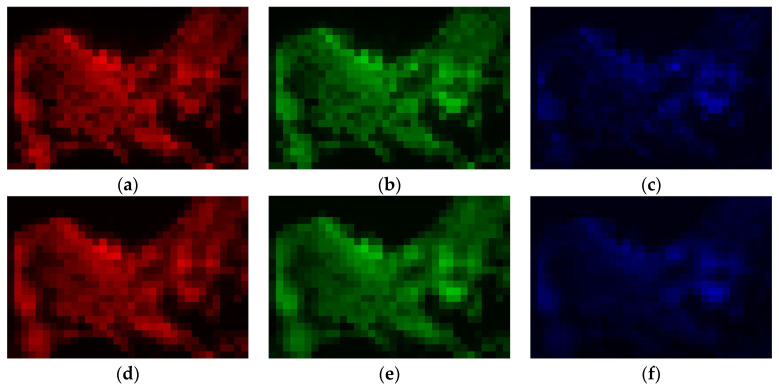
Original vs. CNN-estimated (*k* = 5) RGB levels in *Haifa area*: (**a**) original red; (**b**) original green; (**c**) original blue; (**d**) estimated red; (**e**) estimated green; (**f**) estimated blue. Note: The images are of panchromatic NTL spatial resolution.

**Figure 6:**
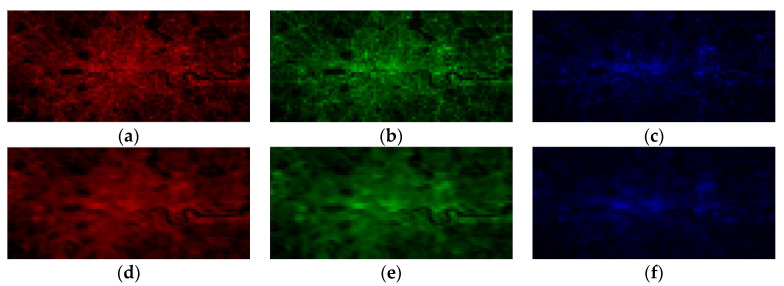
Original vs. CNN-estimated (*k* = 13) RGB levels in *London area*: (**a**) original red; (**b**) original green; (**c**) original blue; (**d**) estimated red; (**e**) estimated green; (**f**) estimated blue. Note: The images are of panchromatic NTL spatial resolution.

Visual inspection witnesses fairly sufficient similarity between the original RGB levels and those restored using the proposed CNN models, although the restored images look slightly more blurred. Figure 5 and Figure 6 report such pairs for R, G, and B bands for two cities, representing small (Haifa, Israel) and large (London, UK) metropolitan areas. 

**Figure 7:**
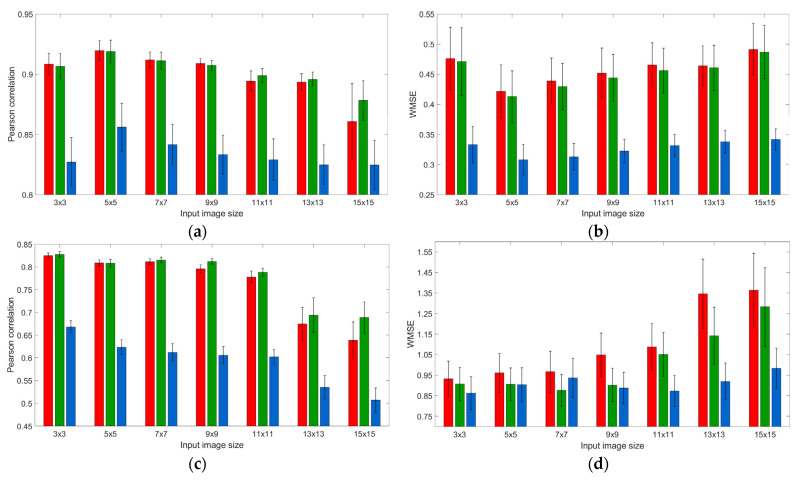
The effects of input image size on CNN performance for the training (top row) and testing (bottom row) datasets, in terms of averaged Pearson’s correlation coefficients (**a**,**c**), and WMSE (**b**,**d**). Notes: In case of Pearson’s correlation (**a**,**c**), greater means better; In case of WMSE (**b**,**d**), lower means better. Error lines stand for the standard error of the mean.

In Figure 7, we report the effect of the input image size upon two performance indicators—Pearson’s correlation and WMSE for each color band, separately for (eight) training and (56, i.e., seven for each of the eight training) testing sets. As one can see from the figures, both indicators’ levels, averaged across *training sets* (Figure 7a,b), changed non-monotonically, with the extremum at 5 × 5 image size, for any color band prediction. To check whether the input size 5 × 5 is indeed the extremum, we conducted a one-tailed paired samples *t*-test for the statistical significance of the difference of means—against both smaller (3 × 3) and larger (7 × 7) input sizes. For all channels, mean levels of Pearson’s correlation for 5 × 5 input image size were significantly higher than the ones of either 3 × 3 and 7 × 7 input image size (|*t*| > 2.335; *p* < 0.026 for the red channel; |*t*| > 2.404; *p* < 0.024 for the green channel; and |*t*| > 2.531; *p* < 0.020 for the blue channel). In the meantime, the pattern in terms of WMSE levels was something less pronounced: 5 × 5 input image size was always significantly better than 3 × 3 image (|*t*| = 3.227; *p* = 0.007 for red channel; |*t*| = 2.812; *p =* 0.013 for green channel; and |*t*| = 3.126; *p =* 0.008 for blue channel), but not always better than 7 × 7 counterpart (|*t*| = 1.228; *p =* 0.130 for red channel; |*t*| = 1.884; *p =* 0.051 for green channel; and |*t*| = 0.962; *p =* 0.184 for blue channel). 

**Figure 8:**
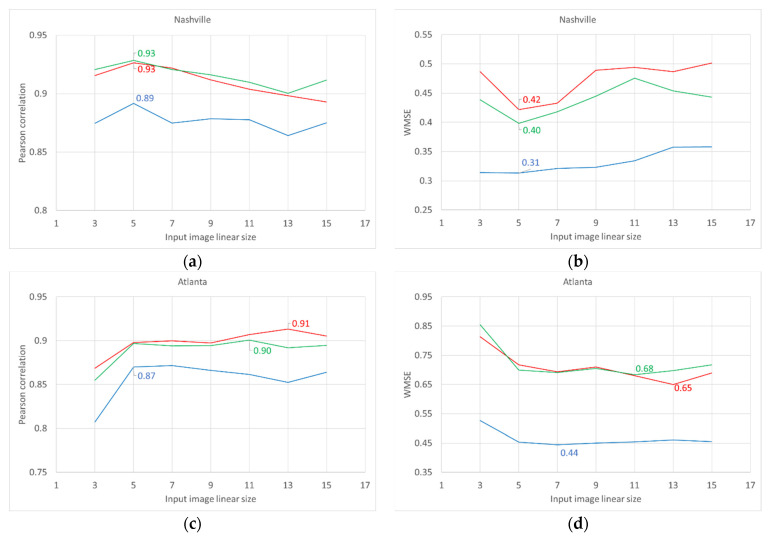
The effects of input image size on the performance of CNN models built for selected training datasets, in terms of Pearson’s correlation coefficients (left panel), and WMSE (right panel). Notes: In the case of Pearson’s correlation (**a**,**c**), greater means better; In the case of WMSE (**b**,**d**), lower means better; the best values are labeled.

We should however notice that—if to analyze the models’ performance indicators for each training dataset separately—optimal input image size is relatively small for smaller, either in terms of area or population, metropolitan areas (5 × 5 image size is optimal for Haifa (with ~0.3 mln people population), Khabarovsk (~0.6 mln people), Nashville (~0.7 mln people), and Naples (~3 mln people)), and larger for bigger metropolitan areas (input images of generally greater than 7 × 7 size are optimal for Atlanta (with ~5.5 mln population), London (~9 mln people), Tianjin (~15 mln people), and Beijing (~21.5 mln people)). Interestingly, for bigger areas, there emerges differentiation in optimal input image size for different color band intensities prediction. Generally, for short-wavelength blue lights, the optimal input image size was smaller than for long-wavelength red lights (for instance, 5 × 5 and 7 × 7 optimal image size for blue lights and 11 × 11 and 13 × 13 for red lights correspondingly in Beijing and Atlanta). In Figure 8 we report models’ performance indicators for two selected training datasets, representing typical relatively small (Nashville–see Figure 8a,b) and big (Atlanta–see Figure 8c,d) metropolitan areas.

We should note that for *testing datasets*, the tendency in the models’ performance indicators is less pronounced (Figure 7c,d), perhaps due to averaging across a greater amount of heterogeneous datasets. Visually, the models generally demonstrate the best performance upon 3 × 3 input images. Although, the difference with 5 × 5 input image size is not always statistically significant. Due to violation of normality, we replaced the paired sample *t*-test for the statistical significance of the difference of means by non-parametric Wilcoxon signed-rank test for the difference of medians. For red and green channels, we found a significant difference in terms of Pearson’s correlation (*p* < 0.001) but not WMSE (*p* > 0.204); In contrast, for the blue channel, the difference appeared significant in terms of WMSE (*p* < 0.001) but not Pearson’s correlation (*p >* 0.801). In the meantime, a more detailed examination shows that models built for smaller metropolitan areas (Haifa, Khabarovsk, Naples, and Nashville) generally fit better other relatively small areas regardless of input image size; While models built for relatively large metropolitan areas (Atlanta, Beijing, London, and Tianjin) fit better other large areas given large enough input image size (not shown). 

### 3.2. CNN Models Comparison with Other Machine Learning Techniques

**Figure 9:**
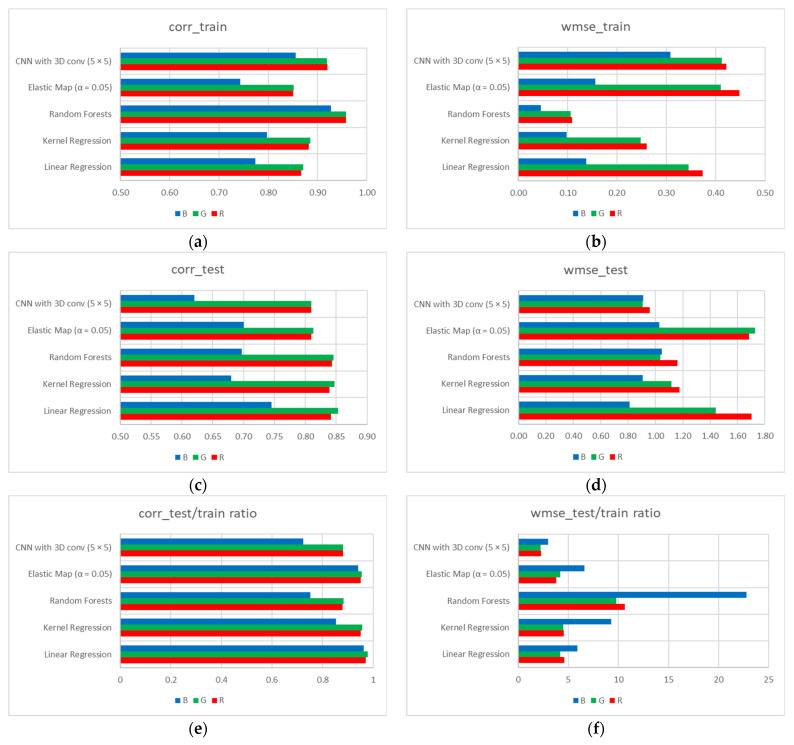
Mutual comparison of linear and kernel regressions, random forest, elastic map, and best-performing CNN models for the training (top row) and testing (bottom row) datasets, in terms of averaged Pearson’s correlation coefficients (**a**,**c**), and WMSE (**b**,**d**). Note: In case of Pearson’s correlation (**a**,**c**), greater means better; In case of WMSE (**b**,**d**), lower means better.

We should also note that the best-performing CNN models (with 5 × 5 input images) demonstrate comparable performance with previously explored techniques [27] in terms of Pearson’s correlations both for training and testing datasets (Figure 9a,c,e), but, compared to other techniques, perform much more stable in terms of WMSE upon training and testing sets—the ratios are within 2.20–2.95 diapason for CNN vs. 3.76–22.78 for other techniques (Figure 9f).

## 4. Discussion and Conclusions

The present study aimed at restoring RGB intensities, reported by the ISS [25], from VIIRS-DNB-provided panchromatic night-time lights imagery [17] and the levels of the built-up area [54], used as a proxy for land-use types. We used corresponding datasets for eight metropolitan areas: Atlanta (US), Beijing (China), Haifa (Israel), Khabarovsk (Russia), London (UK), Naples (Italy), Nashville (US), and Tianjin (China), as case studies. To restore the RGB level of the pixel under analysis, we used panchromatic NTL and built-up area levels from varying neighborhoods, starting from the first-order neighboring pixels and up to the neighbors of the seventh level. Thus, for each of the eight metropolitan areas, we sliced input layers for small-scale squared fragments using a kernel with unit stride, and consequently–for each input image size dataset–run CNN models, using either red, green, or blue light level of the central pixel from the corresponding fragment as a dependent variable. Each model, built for a certain metropolitan area dataset, was validated over the rest seven cities.

As our analysis revealed, for relatively small metropolitan areas, either in terms of area or population (such as Haifa, Khabarovsk, Naples, and Nashville), the best-performing models for any color light band prediction were built for 5 × 5 input image size, while for larger ones (such as Atlanta, Beijing, London, and Tianjin) the optimal input image size was at least 7 × 7 pixels. A speculative explanation is on the average larger physical extent of residential quarters, commercial and entertainment centers, and industrial facilities in larger cities. Yet, this assumption should be explicitly tested. 

Another important finding of the present study is that for bigger metropolitan areas emerges differentiation in optimal input image size for different color band lights prediction. Generally, for long-wavelength red lights, the optimal input image size is larger than for short-wavelength blue lights (compare 11 × 11 and 13 × 13 pixels optimal image size for red lights and 5 × 5 and 7 × 7 pixels for blue lights correspondingly in Beijing and Atlanta). There exist several indications that red and green lights are more associated with residential areas, while industrial and commercial facilities are often lit by blue lights [19,61]. Since residential areas are usually more extensive, while industrial and commercial facilities are localized, red and green lights are expected to be predicted better by larger neighborhoods. In the meantime, this tendency was not confirmed by the case of London (with 9 × 9 and 13 × 13 optimal input image size for red and blue lights, correspondingly) and Tianjin (with the same 7 × 7 optimal input image size for all colored lights prediction) and thus requires further investigation. Other directions of future investigation might include experiments with a larger amount of metropolitan areas and alternative CNN architectures.

To compare different machine learning techniques, none of them demonstrated an absolute advantage over its counterparts: Instead, the advantage depended on the chosen performance indicator. Compared to our previous study, where we examined *first-order neighborhood effect only* and used several machine learning techniques, such as linear regression, non-linear kernel regression, random forest, and elastic map models [27], CNN models performed something worse in terms of Pearson’s correlation upon testing sets. In this sense, it seems perspective to seek for CNN architectures less sensitive to data heterogeneity. At the same time, compared to other machine learning techniques, CNN models showed better performance in terms of WMSE for testing datasets for red (0.91 for CNN models vs. 1.04–1.73 for other machine learning techniques) and green (0.96 for CNN models vs. 1.16–1.70 for other machine learning techniques) bands prediction. We explain such an improvement by accounting for the varying neighborhood effect, which is more important for relatively vaster residential area-associated bands [19,61]. 

## Figures and Tables

**Table 1:** The number of inputs for CNN depending on input image size.

Region	Input Image Size						
	3 × 3	5 × 5	7 × 7	9 × 9	11 × 11	13 × 13	15 × 15
Atlanta	5609	5313	5025	4745	4473	4209	3953
Beijing	5400	5088	4784	4488	4200	3920	3648
Haifa	900	782	672	570	476	390	312
Khabarovsk	3550	3312	3082	2860	2646	2440	2242
London	4850	4560	4278	4004	3738	3480	3230
Naples	1872	1700	1536	1380	1232	1092	960
Nashville	2695	2491	2295	2107	1927	1755	1591
Tianjing	6045	5733	5429	5133	4845	4565	4293

## Data Availability

The data and the code presented in this study are openly available from https://github.com/Mirkes/Sensor-paper-for-CNN-based-image-colouring. (accessed on 2 November 2021).
